# Curcumin Oxidation Is Required for Inhibition of *Helicobacter pylori* Growth, Translocation and Phosphorylation of Cag A

**DOI:** 10.3389/fcimb.2021.765842

**Published:** 2021-12-24

**Authors:** Ashwini Kumar Ray, Paula B. Luis, Surabhi Kirti Mishra, Daniel P. Barry, Mohammad Asim, Achyut Pandey, Maya Chaturvedi, Jyoti Gupta, Shilpi Gupta, Shweta Mahant, Rajashree Das, Pramod Kumar, Shalimar, Keith T. Wilson, Claus Schneider, Rupesh Chaturvedi

**Affiliations:** ^1^ School of Biotechnology, Jawaharlal Nehru University, New Delhi, India; ^2^ Department of Microbiology, Saheed Rajguru College of Applied Sciences for Women, University of Delhi, New Delhi, India; ^3^ Department of Environmental Studies, University of Delhi, New Delhi, India; ^4^ Department of Pharmacology and Vanderbilt Institute of Chemical Biology, Vanderbilt University School of Medicine, Nashville, TN, United States; ^5^ Division of Gastroenterology, Hepatology, and Nutrition, Department of Medicine, Vanderbilt University Medical Center, Nashville, TN, United States; ^6^ Centre for Medical Biotechnology, Amity Institute of Biotechnology, Amity University, Noida, India; ^7^ Department of Chemistry, Sri Aurobindo College, University of Delhi, New Delhi, India; ^8^ Department of Gastroenterology and Human Nutrition Unit, All India Institute of Medical Sciences, New Delhi, India; ^9^ Center for Mucosal Inflammation and Cancer, Vanderbilt University Medical Center, Nashville, TN, United States; ^10^ Veterans Affairs Tennessee Valley Healthcare System, Nashville, TN, United States; ^11^ Special Centre of Systems Medicine, Jawaharlal Nehru University, New Delhi, India; ^12^ Nanofludiks Research Pvt. Ltd. AIC-JNUFI, Jawaharlal Nehru University (JNU) New Delhi, New Delhi, India

**Keywords:** curcumin, metabolic transformation, reduced curcumin, *Helicobacter pylori*, CagA

## Abstract

Curcumin is a potential natural remedy for preventing *Helicobacter pylori*-associated gastric inflammation and cancer. Here, we analyzed the effect of a phospholipid formulation of curcumin on *H. pylori* growth, translocation and phosphorylation of the virulence factor CagA and host protein kinase Src *in vitro* and in an *in vivo* mouse model of *H. pylori* infection. Growth of *H. pylori* was inhibited dose-dependently by curcumin *in vitro*. *H. pylori* was unable to metabolically reduce curcumin, whereas two enterobacteria, *E. coli* and *Citrobacter rodentium*, which efficiently reduced curcumin to the tetra- and hexahydro metabolites, evaded growth inhibition. Oxidative metabolism of curcumin was required for the growth inhibition of *H. pylori* and the translocation and phosphorylation of CagA and cSrc, since acetal- and diacetal-curcumin that do not undergo oxidative transformation were ineffective. Curcumin attenuated mRNA expression of the *H. pylori* virulence genes *cagE* and *cagF* in a dose-dependent manner and inhibited translocation and phosphorylation of CagA in gastric epithelial cells. *H. pylori* strains isolated from dietary curcumin-treated mice showed attenuated ability to induce cSrc phosphorylation and the mRNA expression of the gene encoding for IL-8, suggesting long-lasting effects of curcumin on the virulence of *H. pylori*. Our work provides mechanistic evidence that encourages testing of curcumin as a dietary approach to inhibit the virulence of CagA.

## Introduction

*H. pylori* a is Gram-negative bacterium that colonizes the host stomach of more than half of the world population, making it the most prevalent human pathogen worldwide (Wroblewski et al., 2010; Venerito et al., 2018). *H. pylori* infection initially causes inflammation of the stomach and over time the disease progress through a well define cascade that includes non-atrophic gastritis, multifocal atrophic gastritis, intestinal metaplasia, dysplasia, and gastric carcinoma (Kusters et al., 2006; Correa and Piazuelo, 2012). Gastric adenocarcinoma is the third leading cause of cancer-related death worldwide, and *H. pylori* infection is the most potent known risk factor for this malignancy (Parkin, 2004; Cover, 2016).

Although an NIH consensus conference determined that *H. pylori* is a Class I carcinogen, treatment was recommended only for peptic ulcer patients (Yamada et al., 1994). Since more than half of the human population is infected, universal treatment is not feasible. Furthermore, *H. pylori* may often behave as a commensal organism, making it difficult to predict which individuals will develop cancer. Finally, the concept of the “African, Indian and Colombian Enigma” has emerged based on the observation that the prevalence of *H. pylori* colonization in this area is high, but the frequency of gastric cancer is extremely low, and one of the reasons may be dietary differences (Mathew et al., 2000; Rao et al., 2002; Phukan et al., 2006; Wang et al., 2009; Yassibas et al., 2012). Epidemiological studies and animal models of disease suggest that *H. pylori* causes serious disease, it may be called a pathobiont (Peek, 2004; Amedei et al., 2010; Martin and Solnick, 2014; Von Arnim et al., 2016; Li and Perez-Perez, 2018). Also, there has been an increased prevalence of antibiotic-resistant strains (Graham, 1998; Shao et al., 2018). These observations argue against alternative to antibiotic treatment. New therapeutic interventions are warranted that not only reduce the viability but also decreases the pathogenicity of *H. pylori*.

The African enigma has been attributed to parasitic co-infection in humans, and, it was demonstrated that the geographical origin of the bacteria may also be the basis for the Colombian enigma (Nguyen et al., 2008; Ghoshal et al., 2010). Diet has been postulated as a possible explanation for the Indian enigma. Curcumin, a secondary metabolite of the plant turmeric, is a common food ingredient in India (Zaidi, 2016). The turmeric plant is used in the traditional Indian medicine system, Ayurveda, to treat gastrointestinal diseases including gastric dyspepsia (Thavorn et al., 2014; Zaidi, 2016). Importantly, curcumin modulates several biological functions, including apoptosis, cell proliferation, and immune responses (Ireson et al., 2002). Although curcumin has been shown to reduce the growth of *H. pylori* the low bioavailability and rapid metabolic biotransformation of curcumin have limited its use as a therapeutic agent in animal models of disease (Anand et al., 2007; De et al., 2009; Prasad et al., 2014).

Since biotransformation of curcumin by *H. pylori* has not been studied, we investigated the contribution of reductive and oxidative metabolic pathways to the disposition of *H. pylori* to curcumin. Reduction of curcumin in *E. coli* is catalyzed by CurA, yielding dihydrocurcumin and tetrahydrocurcumin (Hassaninasab et al., 2011). Oxidation of curcumin can occur non-enzymatically in an autoxidative process or enzyme-catalyzed by peroxidases (Schneider et al., 2015b). Either affords the abstraction of phenolic hydrogen resulting in a quinone methide electrophilic intermediate that undergoes oxygen addition and further rearrangement to a bicyclopentadione as the final product (Gordon et al., 2015). Early reactive intermediates of oxidative transformation have been implicated as the mediators of topoisomerase II poisoning, inhibition of IKKβ, and the secretion of glucagon-like peptide-1 by curcumin and here we analyzed their role on the growth inhibition of *H. pylori.* Although curcumin has been shown to reduce the growth of *H. pylori* the low bioavailability have limited its use as a therapeutic agent but phospholipid formulation has been repeatedly shown to increase the bioavailability of curcumin (Cuomo et al., 2011; Franceschi et al., 2016).

Our work here demonstrates the effect of a phospholipid formulation of curcumin on *H. pylori* growth, translocation and phosphorylation of the virulence factor CagA and host protein kinase Src *in vitro* and in an *in vivo* mouse model of *H. pylori* infection.

## Materials and Methods

### Chemicals

Phospholipid formulation of curcumin (which is called “Meriva”) was purchased from sigma Aldrich; pure curcumin was synthesized as described in (Pabon, 1964; Gordon et al., 2013) and was used as a control. Curcumin and its analogues were synthesized following a modified procedure originally developed by Pabon (Pabon, 1964; Gordon et al., 2013). Curcumin has been prepared from vanillin and acetylacetone in the presence of tri-sec. butyl borate and butylamine. The reaction was carried out at room temperature in ethyl acetate. Other compounds were prepared using suitable aldehyde (0.2 mole), tributyl borate (0.4 mole) and the reaction product of acetylacetone (0.1 mole) and boric anhydride (0.07 mole) were dissolved in 100 ml of dry ethyl acetate. Butylamine was added (0.5 ml every 10 min; total 2 ml) while stirring (Pabon, 1964; Gordon et al., 2013).

### Bacterial Cultures

*H. pylori* strains 26695, (Chaturvedi et al., 2015; Jang et al., 2017) PMSS1, (Chaturvedi et al., 2015; Jang et al., 2017) PZ5056G, (Chaturvedi et al., 2015; Jang et al., 2017) and B128 7.13 (Chaturvedi et al., 2015; Jang et al., 2017) were maintained on plates of tryptic soy agar containing 5% sheep blood. Before experiments, bacteria were grown overnight in Brucella broth supplemented with 10% FBS at 37°C (Johnson-Henry et al., 2004). *Escherichia coli strain DH 5-α* and *Citrobacter rodentium* strain ICC168 (Petty et al., 2010) were maintained on LB plates, and starter cultures were grown overnight in LB broth at 37°C with or without agitation, respectively (all the chemicals procured from sigma St. Louis).

### Bacterial Growth Curves

Bacterial growth curves were determined. As 0.1 optical density at 560 nm (OD560) *H. pylori* strains were used to inoculate new Brucella broth, and bacterial growth was monitored by optical density for 24 h. Some cultures were supplemented with synthetic curcumin, (Pabon, 1964) or Meriva, and differences in growth were measured. The effect of Meriva on *Citrobacter rodentium* and *Escherichia coli* was determined in LB broth cultures begun at 0.05 OD560, and bacterial growth was monitored by optical density.

### Quantification of Curcumin and Metabolites by LC-MS Analysis

Samples were extracted using Waters HLB cartridges and dissolved in 50 μl of acetonitrile/water (1:1) for LC-MS analysis. Samples were electro sprayed into a Thermo Vantage triple quadruple mass spectrometer operated in the negative ion mode and coupled to a Waters Acquity UPLC system with a Waters Symmetry Shield C18 3.5 μm column (2.1 x 100 mm) using a gradient of 5% to 95% acetonitrile in water 0.1% formic acid within 3 min and a flow rate of 0.4 μl/min (Luis et al., 2018) (all the chemicals procured from sigma St. Louis).

### Human Gastric Cancer Cell Culture and Infection With *H. pylori*

AGS cells were grown in (DMEM) Advanced Dulbecco’s Modified Eagle’s Medium/F12 medium (sigma) supplemented with 10% FBS and 2 mM glutamine and antibiotics. *H. pylori* were grown for 6 h with or without exposure to curcumin or Meriva, at which time the bacteria were collected and washed with PBS. AGS cells were infected with treated *H. pylori* at a multiplicity of infection (MOI) which was determined of 10. MOI was measured as the number of pathogen that are added per cell during infection. If one million pathogens are added to one million cells, the MOI is one (Bussiere et al., 2006).

### Gene Expression in Epithelial Cells

mRNA Expression of *CXCL8* the gene encoding for IL-8, was measured in AGS epithelial cells exposed to *H. pylori* strains. Total RNA was isolated 6 h after exposure, using TriZOL (Invitrogen), and cDNA was synthesized from 1 µg RNA, as described in kits protocol (Invitrogen). Real-time PCR was performed, and relative gene expression was calculated from second derivative maximum values using β-actin as a reference gene. *CXCL8* primers were *5′-TAGCAAAATTGAGGCCAAGG-3′* and *5′-AAACCAAGGCACAGTGGAAC-3′*.

### mRNA Expression in *H. pylori*

*H. pylori* 26695 was grown for 6 h with or without Meriva. Total RNA was extracted from 10^8^ bacteria using TriZOL (Invitrogen), and cDNA was synthesized from 1 µg RNA (Rokbi et al., 2001). Real-time PCR was performed, and relative gene expression was calculated using 16S rRNA as a reference gene (Rokbi et al., 2001). *cagA* E, M and 16S primers (Barry et al., 2011) were *cagA 5′-ACCAACAAGGTAACAATGTGGC-3′* and *5′-TCGTTGTGAGCCTGTGAGTTGGT-3′*. *cagE 5′-CAATGGGTGGGGAGTATGTC-3′* and *5′-TGCTCCATTGTTGCATTTGT-3′*. *cagM 5′-GGTTGCGTTTGGAGTTTTGTCGGC-3′* and *5′-AGCGTCTTCTTTTGCGGCCACT-3′*. 16S *5′-CAGCTCGTGTCGTGAGATGT-3′* and *5′-CGTAAGGGCCATGATGACTT-3′*.

### Western Blotting and Detection of CagA Translocation

The cells were removed by trypsinization and lysates were prepared in buffer composed of 50 mM Tris-HCl (pH 8.0), 150 mM NaCl, 1% (v/v) NP-40, and 0.1% (w/v) sodium dodecyl sulfate. 20 µg of protein per lane was resolved by electrophoresis on 10% Tris-HCl polyacrylamide gels (Bio-Rad) and transferred overnight onto PVDF and blocked in BSA. Translocation of CagA in gastric epithelial cells was detected by level of phosphorylated (phospho)-CagA. Upon injection, CagA undergoes tyrosine phosphorylation by host kinases, and therefore, in our experiments the translocated CagA was detected by anti-phospho-CagA. Phosphorylated (phospho)-CagA, and β-actin levels as loading controls were detected with 1∶300 diluted anti-phosphotyrosine antibodies (Santa Cruz Biotechnology,PY20), 1∶2000 polyclonal anti-*H. pylori* CagA antibody (Austral Biologicals HPA-5000-4), and 1∶20000 anti-β-actin antibody (Sigma A2228), respectively. In some experiments, *H. pylori* co-cultured AGS cell lysates were also probed for levels of phosphorylated-cSrc and cSrc proteins with mouse anti-phospho-cSrc (Y418, dilution 1:300; cell signaling), and mouse anti-cSrc (dilution 1:500; cell signaling), respectively.

### Gastroid Culture and Co-Infection With *H. pylori*

Mouse stomach glands were isolated as described (Wroblewski et al., 2014) by ligating at the esophago-gastric and gastro-duodenal junctions. Released glands were plated in Matrigel (BD Biosciences) containing N-acetylcysteine (1 µM), gastrin (10 nM), epidermal growth factor 10 (50 ng/mL), R-spondin 1 (500 ng/mL), Noggin (100 ng/mL), fibroblast growth factor 10 (100 ng/mL), rWnt3A (100 ng/mL), and Y27632 (10 µM) as described (Shibata et al., 2017) (DMEM)**/**F12 medium supplemented with B27, N2, penicillin/streptomycin, N-2-hydroxyethylpiperazine-N-2-ethane sulfonic acid (10 mM) and Glutamax (2 mM) was overlaid on the Matrigel.

The *H. pylori* were grown in Brucella broth with 10% fetal bovine serum for 6 h with and without Meriva, harvested and microinjected into the lumen of gastroids at a multiplicity of infection of 100:1 (Bussiere et al., 2006). Infected gastroids were cultured for 4 hr in Advanced DMEM/F12 medium without penicillin/streptomycin. Some of the wells were fixed in 4% formaldehyde and embedded in paraffin blocks. Five μM thin sections were cut, and gastroids were stained with H&E and also for PAS-AB as described(Shibata et al., 2017) Some of the gastroids were also processed for immunofluorescence.

### Immunofluorescence

Gastroids were cultured on MatTek dishes (MATTEK Corporation) and stained for phospho-cSrc. In brief, gastroids were washed twice in 1X PBS, and formalin-fixed. Cells were permeabilized using 1X PBS containing 0.1% Triton X-100 (30 minutes, room temperature), and washed three times in 1X PBS. Gastroids were stained with mouse anti-phospho-cSrc (Y 418: Cell Signaling) followed by Alexa Fluor 488-conjugated anti-mouse IgG antibody (1:200, Molecular Probes). Nuclei were stained with DAPI (4 μg/mL). Images were captured using an Olympus FV-1000 Inverted Confocal Microscope. Image acquisition was performed using Fluoview FV10-ASW 1.7 software.

### Mouse Infection

Male C57BL/6 mice aged 6–8 weeks were infected as described (Arnold et al., 2011) *H. pylori* was grown for 2 days on serum plates, harvested, and suspended in brucella broth, and the final concentration was adjusted to 10^7^bacteria/200 μl. Mice were inoculated orogastrically with 0.2 ml of bacterial suspension (10^7^bacteria) (Arnold et al., 2011). Briefly, *H. pylori* PMSS1, a clinical isolate, was grown for 6 h with or without Meriva, and 2×10^7^ bacteria were delivered intra-gastrically on days 0, 2 and 4. After one month, mice were sacrificed (Conlee et al., 2005), and colonization was determined by serial dilution and plating of gastric homogenates. After one week, colonies were counted to determine bacterial load, and single colonies were stored as output strains (D’costa et al., 2018). AGS cells were also co-cultured with output strains from the mouse infection experiment, and cell lysates were probed for levels of phospho-cSrc and cSrc. This study was carried out following recommendations in the Guide for the Care and Use of Laboratory Animals of the National Institutes of Health. The Institutional Animal Care and Use Committee of Vanderbilt University and the Institutional Animal Care and Use Committee of Jawaharlal Nehru University New Delhi approved the protocol (protocol No M/12/046).

### Statistical Analysis

Data are expressed as means ± standard error. Student’s *t* test was used for pairwise comparisons. Data from more than two groups were analyzed by ANOVA, followed by the Newman-Keuls *post hoc* multiple comparisons tests.

## Results

### Phospholipid Encapsulation of Curcumin (Meriva) Enhances Growth Inhibition of *H. pylori*

**Figure 1:**
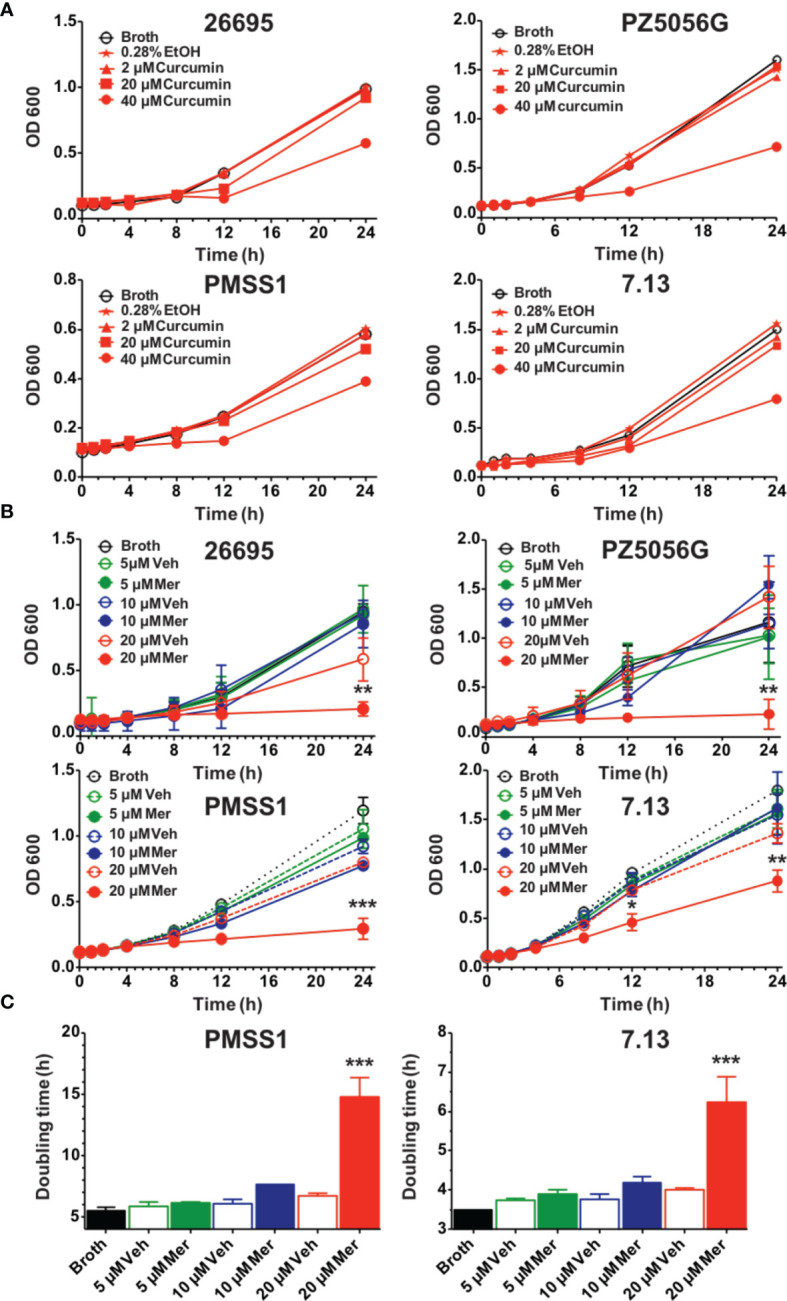
Effects of curcumin and phospholipid encapsulated curcumin (Meriva) on growth of four prototype strains of *H. pylori*. **(A)** Four strains of *H. pylori* (26695, PZ5056G, PMSS1, and 7.13) (0.1 OD) were grown in the presence of vehicle (ethanol) or increasing concentrations of curcumin, and growth was determined at the indicated time points. **(B)** Growth curve of *H. pylori* strains 26695, PZ5056G, PMSS1, and 7.13 cultured in the presence of a phospholipid formulation of curcumin (Meriva, Mer) or vehicle control (Veh, ethanol). **(C)** Generation time of *H. pylori* PMSS1 and 7.13 as determined from the growth curves (**P value < 0.005 ***P value < 0.005).

Curcumin, a non-antibiotic compound is effective against *H. pylori* without any toxicity and resistance (Sarkar et al., 2016). Since the bioavailability of pure curcumin is low its ability to inhibit growth of *H. pylori* was compared to a phospholipid complexed formulation (Meriva) with improved bioavailability and pharmacokinetics (Cuomo et al., 2011; Franceschi et al., 2016). To compare the effects on *H. pylori* growth four prototypic strains were selected, 26695, PZ5056G, PMSS1, and 7.13. All *H. pylori* strains were partially inhibited at 40 μM curcumin resulting in a modest increase in generation time, whereas 20 μM did not show an effect (**Figures 1A, C**). Meriva caused a significant decrease of the growth of all four *H. pylori* strains at 20 μM (**Figure 1B**). This concentration of Meriva also increased the generation time of two representative strains of *H. pylori* at 17.4 h for PMSS1 and at 6.5 h for 7.13 (**Figure 1C** and **Supplementary Figure 1**). We observed Meriva inhibit the growth of *H. pylori* at low concentration, which indicates it is more toxic than curcumin for *H. pylori (* Kidd, 2009 *).* Quantification of curcumin inside the bacteria revealed that the levels were significantly higher in bacteria treated with Meriva (18.6 ± 2.4 ng/10^8^ bacteria) compared to control curcumin (0.5 ± 0.1 ng/10^8^ bacteria) when a concentration equivalent to 20 μM curcumin was applied (**Supplementary Figure 2**) (Blanchard and Nedrud, 2012). This suggested an increase in bioavailability of curcumin upon encapsulation with phospholipids, and this could explain the more growth inhibitory effect compared to pure curcumin.

### Reductive Metabolism of Curcumin Inhibits Its Antimicrobial Effect

**Figure 2:**
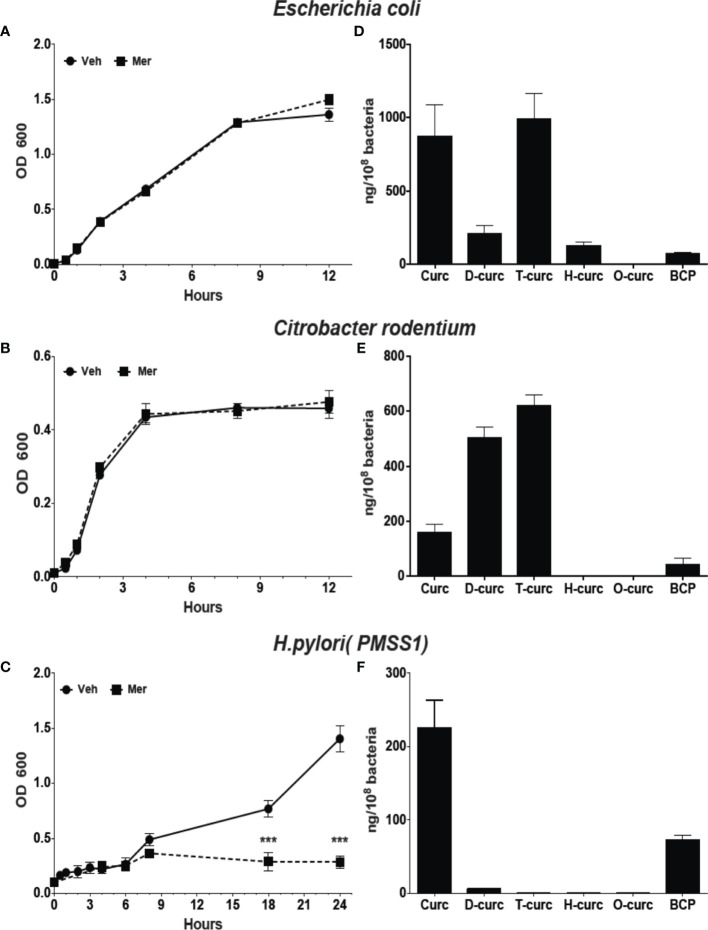
Effect of Meriva on growth of *E. coli*, *Citrobacter rodentium*, and *H. pylori*, and intracellular accumulation of curcumin and its derivatives. **(A–C)** Bacteria were grown in Brucella broth containing 20 µM Meriva or vehicle (ethanol), and growth was monitored by optical density for indicated time. **(D–F)** Bacteria were harvested and the concentration of curcumin, dihydro-(D-curc,DHC)-, tetrahydro-( T-curc-THC)-, hexahydro-(H-curc,HHC)- and octahydro-(O curc,OHC)-curcumin as well as bicyclopentadione (BCP) was quantified using LC-MS. ***P ≤ 0.001.

Metabolism affects the biological activity of curcumin with reduction of the double bonds as well as conjugation decreasing activity (Anand et al., 2007). Reduction of curcumin in *E. coli*, is catalyzed by the enzyme NADPH-dependent curcumin reductase (CurA) yielding dihydro-curcumin and tetrahydrocurcumin (Hassaninasab et al., 2011). Phylogenetic analysis showed the widespread presence of CurA in enteric bacteria (**Supplementary Figure 3**). To understand the effect of reductive metabolism we studied the effect of Meriva on the growth of *H. pylori*, *E. coli*, and the enteropathogenic rodent equivalent, *Citrobacter rodentium*. The growth of *E. coli* and *Citrobacter rodentium* was not inhibited compared to *H. pylori* at 20 μM Meriva (**Figures 2A–C**). We detected high levels of intracellular curcumin and the reduced metabolites, dihydro-curcumin and tetrahydro-curcumin, in *E. coli* and *Citrobacter rodentium* (**Figures 2D, E**), while only curcumin was detected in *H. pylori* (**Figure 2F**). This indicated that *H. pylori* was not able to metabolize curcumin by reduction, and suggested that, conversely, reductive metabolism of curcumin enabled *E. coli* and *Citrobacter rodentium* to evade its antibacterial effect.

**Figure 3:**
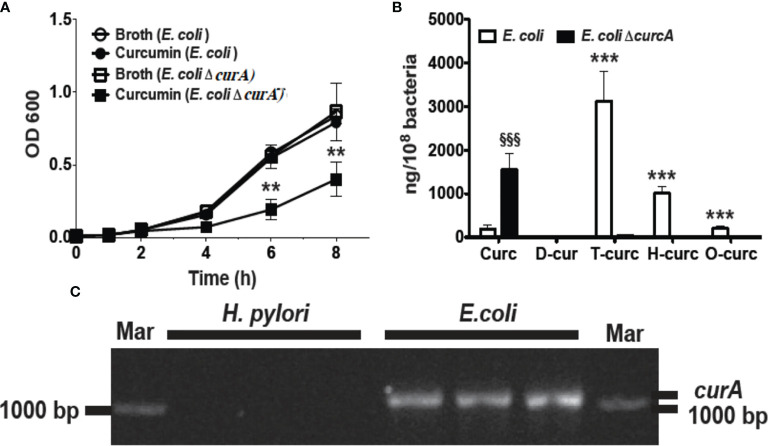
Deletion of *curA* gene in *E. coli* results in growth inhibition by curcumin. **(A)** Growth curve of *E. coli* and its deletion mutant (Δ*curA*) in the presence of curcumin or vehicle (**P value < 0.005 ***P value < 0.005). The deletion of curA was done by PCR splicing method in which a pair of primers flanking the region where the deletion to be made, two complementary primers comprising a region of -15 bp to +15 bp related to the junction point were used and a high fidelity polymerase was used for PCR. **(B)** Quantification of curcumin and its reduced metabolites dihydro-curcumin (D-curc), tetrahydro-curcumin (T-curc), hexahydro-curcumin (H-curc), and octahydro-curcumin (O-curc) in *E. coli* and its *curA* deletion mutant. **(C)** RT-PCR analysis of *curA* expression in genomic DNA isolated from *H. pylori* or *E. coli*. ^§§§^P ≤ 0.001.

In order to analyze the role of CurA in the reduction of curcumin and evasion of growth inhibition, we generated an isogenic deletion mutant of *curA* in *E. coli* strain DH5 -α (*E. coli* Δ*curA*).The deletion of curA was done by PCR splicing method in which a pair of primers flanking the region where the deletion to be made, two complementary primers comprising a region of -15 bp to +15 bp related to the junction point were used and a high fidelity polymerase was used for PCR. The transformed *E. coli* were used for the assay. Curcumin inhibited the growth of the *E. coli* Δ*curA* deletion mutant to a similar extent as observed for *H. pylori* (**Figure 3A**). The reduced metabolites dihydro- and tetrahydro-curcumin were absent in the *curA* deletion mutant despite high levels of curcumin in the cells (**Figure 3B**) while wild type *E. coli* mainly contained the reduced metabolites tetra- and hexahydro-curcumin and negligible amounts of curcumin (**Figure 3B**). PCR analysis (Hassaninasab et al., 2011) of genomic DNA confirmed the presence of the CurA reductase in *E. coli* DH5-α and showed absence of the gene in *H. pylori* (**Figure 3C**). The NCBI BLAST (Default parameter) showed no hits against *H. pylori*. We also checked the curA in 18 clinical isolates with *E. coli* as positive control (**Supplementary Figure 6**). CurA gene was not detetected in any of the isolates. These data strongly suggested a role of CurA and reductive metabolism in evading growth inhibition by curcumin in enteric bacteria.

### Oxidative Transformation of Curcumin Is Required for Anti-*H. pylori* Effect of Meriva

**Figure 4:**
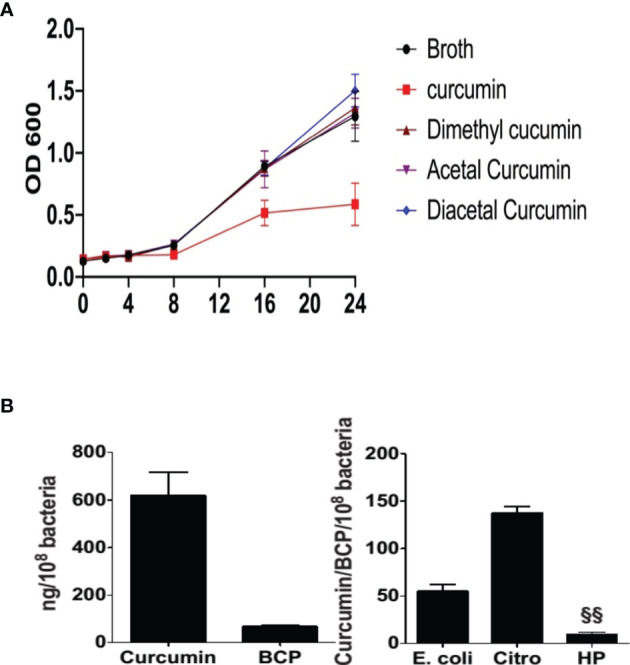
Effect of curcumin and its stable analogs on growth of *H. pylori* and intracellular accumulation of bicyclopentadione (BCP) in *H. pylori*, *E. coli, and Citrobacter rodentium* **(A)** *H pylori* was grown in the absence or presence of curcumin, dimethyl-, acetal-, or diacetal-curcumin, and growth was determined by measuring optical density. **(B)** Intracellular accumulation of curcumin and BCP in *H. pylori.* **(C)** After 24hr treatment the ratio of accumulation of curcumin and BCP in *E. coli, Citrobacter rodentium* (Citro), and *H. pylori* (Hp). ^§§^P ≤ 0.01.

Reductive metabolism of curcumin has been shown to limit its biological effects (Ireson et al., 2002), whereas oxidative transformation appears to be required to affect protein targets with redox-active cysteine residues (Ketron and Osheroff, 2014; Schneider et al., 2015). Since reductive metabolism decreased growth inhibition, we next determined the role of oxidative metabolism of curcumin. To accomplish that, we compared curcumin to its derivatives, diacetalcurcumin, acetalcurcumin, and 4’,4”-dimethylcurcumin. The latter three do not or not readily undergo oxidative transformation (Edwards et al., 2017). Diacetalcurcumin and acetalcurcumin, similar to reduced curcumin, did not inhibit the growth of *H. pylori* at 40 μM concentration, whereas the same concentration of curcumin inhibited growth significantly (**Figure 4A**). Growth inhibition by curcumin was associated with the formation of bicyclopentadione (BCP), the most abundant stable end product of oxidative transformation, (Luis et al., 2017) that was present in about 10% abundance relative to curcumin inside the bacteria (**Figure 4B**). The curcumin/BCP ratio was significantly higher in *E. coli* and *Citrobacter rodentium* than in *H. pylori* (**Figure 4C**), indicating a greater contribution of the oxidative pathway to the metabolism in *H. pylori*. This was consistent with the hypothesis that growth inhibition is dependent on oxidative transformation of curcumin.

### Curcumin Reduces cagA Translocation and Phosphorylation

**Figure 5:**
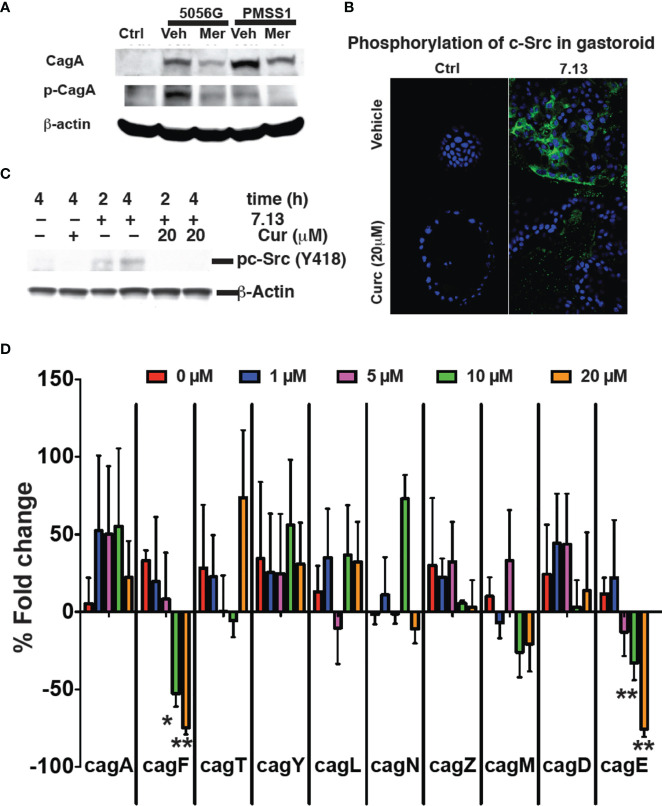
Curcumin prevents cagA phosphorylation. **(A)** Gastric epithelial cells were infected with *H. pylori* strains PZ5056G or PMSS1 in the absence or presence of Meriva (20 μM). CagA and phospho-CagA were determined by Western blotting using β-actin as loading control. Representative results from three independent experiments are shown. **(B)** Mouse stomach glands were isolated and plated in matrigel in the presence of growth factors. *H. pylori* was grown for 6 h with or without Meriva, harvested, and microinjected into the lumen of the gastroids. Infected gastroids were cultured and stained using an anti-phospho-cSrc antibody followed by Alexa Fluor 488-conjugated anti-rabbit IgG antibody (green). Nuclei were stained with DAPI (blue). **(C)** Western blot analysis of c-Src phosphorylation in stomach organ cultures used in panel **(B)**. **(D)** Q-PCR analysis (% fold change) of the expression of the genes encoding for the Cag proteins A, F, T, Y, L, N, Z, M, D, and E in *H. pylori *(Shaffer et al., 2011; Frick-Cheng et al., 2016) treated with different concentrations of Meriva. *P ≤ 0.05; **P ≤ 0.01.

*H. pylori* translocates the bacterial protein CagA into infected gastric epithelial cells using a type IV secretion system, encoded by the cag pathogenicity island (Odenbreit et al., 2000). Translocation of CagA initiates numerous signaling events that contribute to *H. pylori* pathogenesis. CagA translocation and its subsequent phosphorylation occur in a time dependent manner (Backert et al., 2000). When *H. pylori* was grown in 20 μM Meriva for 6 h and then transferred to only broth, growth recovered to the same rate as for untreated bacteria within the next 24 h. When *H. pylori* were grown in the presence of Meriva for 12 h, bacteria did not recover when transferred to only broth (**Supplementary Figure 4**), indicating that exposure of *H. pylori* to curcumin for 6 h resulted in reversible growth inhibition (Barry et al., 2011). We analyzed early events occurring during reversible growth inhibition. Treatment of *H. pylori* strains PZ5056G and PMSS1 with curcumin for 6 h resulted in reduced translocation of CagA into infected gastric epithelial cells and subsequent phosphorylation (**Figure 5A**).

We prepared gastroids from mouse stomach as described previously in order to test a physiologically more relevant model (Wroblewski et al., 2014; Pompaiah and Bartfeld, 2017). Gastroids were stained with H&E to confirm the morphology and PAS-AB to confirm mucin production as a marker of a functionally viable model (**Supplementary Figure 5**). To analyze the effect of Meriva on the ability of *H. pylori* to activate biological signaling in gastroid epithelial cells, we microinjected *H. pylori* grown for 6 h in control medium or in medium supplemented with 20 μM Meriva. Microinjection of *H. pylori* grown in control broth caused phosphorylation of c-Src as detected by immunofluorescence (**Figure 5B**). Phosphorylation of c-Src was reduced in gastroid epithelial cells microinjected with *H. pylori* grown in the presence of Meriva. We confirmed the immunofluorescence data by Western blotting showing reduced phosphorylation of c-Src in gastroid lysates from *H. pylori* treated with Meriva (**Figure 5C**). *H. pylori* virulent strains encode the *cag* (cytotoxin-associated genes) pathogenicity island (cagPAI). The cagPAI expresses a type IV secretion system (T4SS). This T4SS injects virulence factors such as the CagA effector protein into host target cells. This is achieved by a number of T4SS proteins (Barrozo et al., 2013) and to assess the effect of curcumin on Cag PAI-associated genes (Ikenoue et al., 2001) we performed quantitative analysis for the levels of mRNA of *cag* A, F, T, Y, L, N, Z, M, D, and E. Curcumin dose-dependently decreased levels of *cagF* and *cagE* mRNA, which is a virulence factor for translocation of CagA (**Figure 5D**). It has been reported that both the genes are not co-transcribed (Ta et al., 2012). There might be possible both the genes are involved in virulence and upon treatment with curcumin both the genes get downregulated, the further investigation need to be done.

### Oxidation of Curcumin Is Required to Attenuate Host Response to *H. pylori* Infection

**Figure 6:**
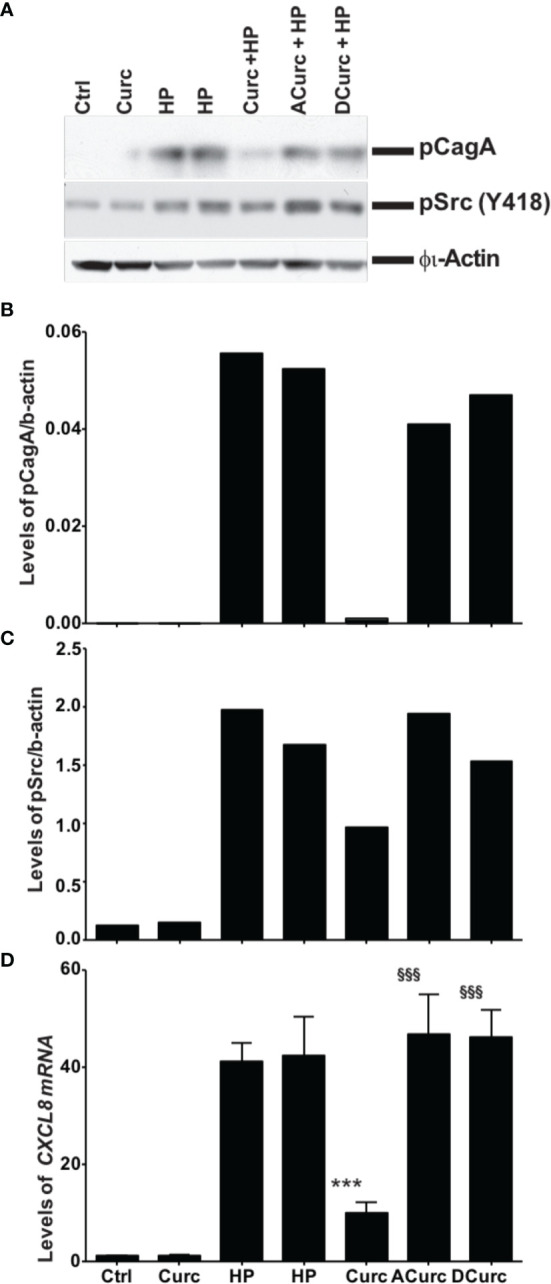
Inhibition of phosphorylation of cagA and Src requires oxidative transformation of curcumin. **(A)** Western blot analysis of CagA and Src phosphorylation in gastric epithelial cells upon infection with *H. pylori* treated with curcumin or its stable analogs acetal-curcumin (ACurc) and diacetalcurcumin (DCurc). The blot was probed with anti-phosphoCagA (top) or anti-phosphoSrc antibody (below), and β actin was used as loading control. Representative results from three independent experiments are shown. **(B, C)** Quantification of Western blotting results described in **(A)**. **(D)** Quantification of *CXCL8* mRNA level in gastric tissue after infection with *H. pylori* treated with curcumin and its stable analogs. ***P ≤ 0.001, ^§§§^P ≤ 0.001.

We compared the effects of curcumin, acetalcurcumin, and diacetalcurcumin on the ability of *H. pylori* to translocate CagA inside host epithelial cells. Translocation was determined *via* the levels of phosphorylated CagA inside infected gastric epithelial cells. Treatment of *H. pylori* with curcumin resulted in near complete inhibition of CagA phosphorylation. This effect was attenuated when *H. pylori* was grown in acetalcurcumin or diacetalcurcumin such that there was no difference in CagA phosphorylation compared to vehicle (**Figures 6A, B**). Treatment of *H. pylori* with curcumin also decreased the levels of phosphorylated c-Src in gastric epithelial cells, and this decrease was not observed by treatment with acetalcurcumin or diacetalcurcumin (**Figures 6A, C**). Levels of phosphorylated CagA have been shown to correlate with the levels of interleukin-8 (IL-8) since phosphorylation induces cellular responses, including IL-8 secretion. Consistent with the results on CagA and c-Src phosphorylation, *H. pylori* grown in the presence of curcumin reduced the levels of *CXCL8* mRNA in gastric epithelial cells whereas the levels induced by *H. pylori* grown in the presence of acetal- or diacetalcurcumin were comparable to vehicle control (**Figure 6D**). These findings are consistent with a role of oxidative transformation of curcumin on the growth of *H. pylori* and its ability to regulate host cell responses.

### *H. pylori* Isolated From Curcumin Treated Mice Induces Attenuated Host Response

**Figure 7:**
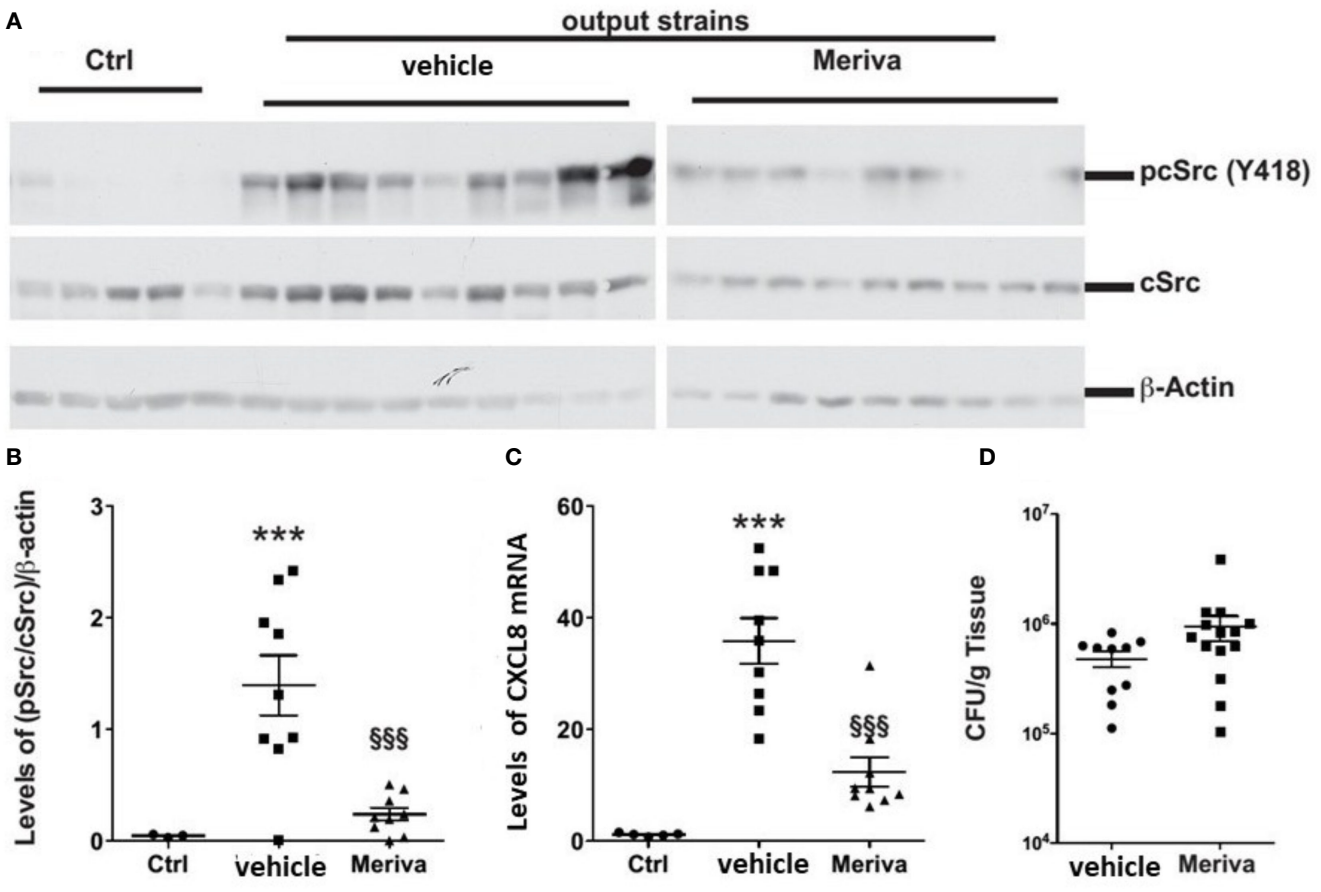
*H. pylori* output strains isolated from dietary curcumin-treated mice induce attenuated host response. **(A)** Western blot analysis of cSrc phosphorylation in AGS cells treated with *H. pylori* isolated from mice fed control chow (vehicle) or chow containing 1% Meriva. *H. pylori*-infected mice fed with chow containing 1% Meriva for 4 weeks shows attenuation of phosphorylation of c-Src. **(B)** Quantification of p-Src/cSrc in the Western blot samples in **(A)** showing attenuation. **(C)** Quantification of *CXCL8* mRNA when co-cultured with AGS cells showing reduced level. **(D)** CFU per gram of tissue showing no colonization. ***P ≤ 0.001, ^§§§^P ≤ 0.001.

We treated *H. pylori*-infected mice with chow containing 1% Meriva for 4 weeks. The output *H. pylori* strains isolated from Meriva-treated mice attenuated phosphorylation of c-Src (**Figures 7A, B**) and reduced levels of *CXCL8* mRNA expression when co-cultured with AGS cells (**Figure 7C**). There was no difference in colonization or inflammation in the stomach of animals treated with vehicle or 1% Meriva (**Figure 7D**).

## Discussion

A major factor in the growth inhibition of *H. pylori* by curcumin was an inability of the bacteria to efficiently detoxify curcumin *via* metabolic reduction of its double bonds. Intestinal bacteria that efficiently reduced curcumin, for example, *E. coli* and *Citrobacter rodentium*, were able to evade the growth inhibitory effect of curcumin. Metabolic reduction was likely achieved by the reductase CurA that was present in *E. coli* and *Citrobacter rodentium*, but absent in *H. pylori*. Genetic deletion of CurA form *E. coli* conferred a similar cytostatic effect of curcumin as was observed with *H. pylori*. similar to the loss of biological activity upon reduction of curcumin in mammalian cells (Huang et al., 1995; Pan et al., 2000; Ireson et al., 2001; Sandur et al., 2007; Ryu et al., 2008). Therefore, metabolic reduction may represent an efficient bacterial strategy to evade antimicrobial effects of curcumin.

While reductive metabolism yields products with less biological activity there is increasing evidence that oxidative transformation of curcumin may be a key event contributing to biological activity (Schneider et al., 2015). One mechanism by which oxidative transformation may mediate biological effects is through binding of reactive intermediates of oxidative transformation to proteins thereby modifying function (Schneider et al., 2015). The reaction intermediates of oxidative transformation of curcumin have electrophilic chemical groups like quinone methide or spiroepoxide moieties that are predicted to bind to nucleophilic cysteine residues on proteins resulting in a covalent modification (Gordon et al., 2015; Luis et al., 2018). For example, curcumin inhibited human topoisomerase IIα *in vitro* whereas the non-oxidizable analog, 4’,4”-dimethyl-curcumin, was inactive (Ketron et al., 2013). Mutation of a critical cysteine residue in the topoisomerase active site eliminated the inhibitory activity of curcumin, identifying this residue as a possible target of curcumin metabolites (Ketron et al., 2013). In a more extensive study the ability of 12 chemically prepared curcumin analogs to undergo oxidative transformation was compared to their potency of inhibiting NF-κB. There was a strong positive correlation between oxidizability and anti-inflammatory activity *via* inhibition of NF-κB (Edwards et al., 2017). The final oxidative metabolite, bicyclopentadione, was inactive in both models, consistent with a role of reaction intermediates. We used a similar approach with curcumin analogs that do not undergo oxidative transformation, and we likewise showed that stable analogs (in which the abstraction of a phenolic hydrogen is blocked by chemical modification) did not inhibit growth of *H. pylori*. While our study tested a limited number of compounds, the results are compatible with a role of oxidative transformation, and consistent with examples of loss of activity upon reductive metabolism of curcumin.

Whether protein binding by oxidative metabolites contributed to growth inhibition of *H. pylori* by curcumin is unclear. Our comparative analyses of chemically synthesized curcumin and Meriva, a phospholipid formulation of curcumin, imply that accumulation of curcumin inside the bacteria played a role in growth inhibition. The phospholipid formulation increases bioavailability of curcumin in animals and humans, and we detected also more curcumin inside the bacteria when Meriva was used compared to curcumin (Marczylo et al., 2007; Cuomo et al., 2011). Curcumin has been reported to affect growth of *H. pylori* by inhibiting shikimate dehydrogenase, a key enzyme in the biosynthesis of aromatic amino acids (Han et al., 2006). There is no evidence of redox regulation of shikimate dehydrogenase or for a role of cysteine residues in the active site of the enzyme (Peek and Christendat, 2015). It is therefore unlikely that reactive oxidative metabolites of curcumin play a role in this inhibitory mechanism. Curcumin has also been shown to protect cultured cells and mice from *H. pylori* infection by inhibiting the activity of matrix metalloproteinases MMP-3 and MMP-9 (Kundu et al., 2011). Redox regulation of MMP activity is well documented (Fu et al., 2001; Nelson and Melendez, 2004; Okamoto et al., 2004; Pei et al., 2006), and curcumin may inhibit MMP activity indirectly by its electrophilic reaction intermediates formed during oxidative transformation.

Besides mediating growth inhibition, curcumin also reduced virulence of *H. pylori*. This was achieved by reducing the translocation of the CagA complex into infected epithelial cells and by reducing its phosphorylation. This went hand-in-hand with reduced phosphorylation of c-Src of the infected host cells and decreased expression of CXCL8 in AGS cells, both effects appeared to involve oxidative transformation since they were abolished when the unoxidizable analogs acetal- and diacetal-curcumin were used. Curcumin ablated the ability of the bacteria to infect human cells and also ablated the pro-inflammatory response of the infected cells.

A further potentially therapeutic mechanism of curcumin was its ability to decrease virulence of *H. pylori* permanently. When *H. pylori* was isolated from mice fed a diet containing Meriva (1%) the bacteria caused reduced phosphorylation of c-Src and decreased expression of CXLC8 in infected gastric epithelial cells compared to bacteria from animals fed the control diet. This indicated that *H. pylori* had become less virulent although there was no difference in the number of bacteria colonizing the stomach of infected animals. Elucidation of the underlying mechanism may require genomic sequencing of the bacteria in order to identify permanent genetic or epigenetic changes as a result of treatment with curcumin.

Taken together, we have identified a number of mechanisms by which curcumin is predicted to result in an overall improved therapeutic outcome on gastric inflammation and carcinogenesis induced by *H. pylori*. Metabolic transformations were identified as key factors in mediating the disposition of *H. pylori* to curcumin. Our results provide an incentive for testing Meriva or any other enhanced bioavailability formulation in future clinical trials of the effects of curcumin on gastric inflammatory diseases induced by *H. pylori*.

## Data Availability Statement

The original contributions presented in the study are included in the article/**Supplementary Material**. Further inquiries can be directed to the corresponding author.

## Ethics Statement

The animal study was reviewed and approved by The Institutional Animal Care and Use Committee of Vanderbilt University and the Institutional Animal Care and Use Committee of Jawaharlal Nehru University New Delhi.

## Author Contributions

AR study concept and design, acquisition of data, analysis and interpretation of data, drafting of the manuscript, statistical analysis. SKM study concept and design, acquisition of data, analysis and interpretation of data, drafting of the manuscript, statistical analysis, acquisition of data. AP, MC, JG, DB, MA, and SG data collection. PK, Shalimar material support, critical revision of manuscript for intellectual content. RC study concept and design, analysis and interpretation of data, drafting of the manuscript, critical revision of manuscript for important intellectual content, statistical analysis, obtained funding, study supervision. PL acquisition of data, analysis and interpretation of data. CS writing and critical revision of manuscript. KW material support, writing and critical revision of the manuscript. All authors contributed to the article and approved the submitted version.

## Funding

Supported in part by National Institutes of Health, USA grants AT 007324 (RC), AT006896 (CS), and P01CA028842 (KW). NHLBI and Fogarty International Centre (FIC), USA grant D43TW009345 and Ref. No./IoE/2021/12/FRP (AR), CSIR (AP,JG,SG), ICMR (MC). PL was supported by a postdoctoral fellowship award from the American Heart Association (16POST27250138). Mass spectrometric analyses were performed in part through Vanderbilt University Medical Center’s Digestive Disease Research Center supported by NIH grant P30DK058404 Core Scholarship.

## Conflict of Interest

Author RC was employed by company Nanofludiks Research Pvt. Ltd.

The remaining authors declare that the research was conducted in the absence of any commercial or financial relationships that could be construed as a potential conflict of interest.

## Publisher’s Note

All claims expressed in this article are solely those of the authors and do not necessarily represent those of their affiliated organizations, or those of the publisher, the editors and the reviewers. Any product that may be evaluated in this article, or claim that may be made by its manufacturer, is not guaranteed or endorsed by the publisher.
