# Supplementary material for: Curcumin Oxidation Is Required for Inhibition of Helicobacter pylori Growth, Translocation and Phosphorylation of Cag A
Source: Front Cell Infect Microbiol. 2021 Dec 24;11:765842. doi: 10.3389/fcimb.2021.765842 (PMC8740292; doi:10.3389/fcimb.2021.765842)
Supplement: Supplementary file 1 [file DataSheet_1.docx]

**Supplementary Figures**

**Curcumin Oxidation is Required for Inhibition of *Helicobacter pylori* Growth, Translocation and Phosphorylation of Cag A**

Ashwini Kumar Ray^1,2 9^, Paula B. Luis^3^, Surabhi Kirti Mishra^1^, Daniel P. Barry^4^, Mohammad Asim^4^, Achyut Pandey^1^, Maya Chaturvedi^1^, Jyoti Gupta^1^, Shilpi Gupta^1^, Shweta Mahant^8^,

Rajashree Das^8^ Pramod Kumar^5^, Shalimar^10^, Keith T. Wilson^4,6,7^, Claus Schneider^3^, Rupesh Chaturvedi^1,11,12*^

^1^School of Biotechnology, Jawaharlal Nehru University, New Delhi, 110067, India.

^2^Department of Microbiology, Saheed Rajguru College of Applied Sciences for Women, University of Delhi, New Delhi, 110096, India.

^3^Department of Pharmacology and Vanderbilt Institute of Chemical Biology, Vanderbilt University School of Medicine, Nashville, TN 37232, U.S.A.

^4^Division of Gastroenterology, Hepatology, and Nutrition, Department of Medicine, Vanderbilt University Medical Center, Nashville, TN 37232, U.S.A.

^5^Department of Chemistry, Sri Aurobindo College, University of Delhi, New Delhi, 110017, India.

^6^Center for Mucosal Inflammation and Cancer, Vanderbilt University Medical Center, Nashville, TN 37232, U.S.A.

^7^Veterans Affairs Tennessee Valley Healthcare System, Nashville, TN 37212, U.S.A.

^8^Centre for Medical Biotechnology, Amity Institute of Biotechnology, Amity University, Noida, UP, India

^9^Department of Environmental studies, University of Delhi, New Delhi, India-110007

^10^ Department of Gastroenterology and Human Nutrition Unit, All India Institute of Medical

Sciences, New Delhi, India,

^11^ special centre of systems medicine, Jawaharlal Nehru University, New Delhi, India.

^12^ Nanofludiks Research Pvt. Ltd. AIC-JNUFI, JNU New Delhi, New Delhi, India.

^∗^ Corresponding author at:

Add: School of Biotechnology, Jawaharlal Nehru University, New Delhi, India

E-mail address: rupesh.chaturvedi.jnu@gmail.com

**A**

**B**

**C**

**D**


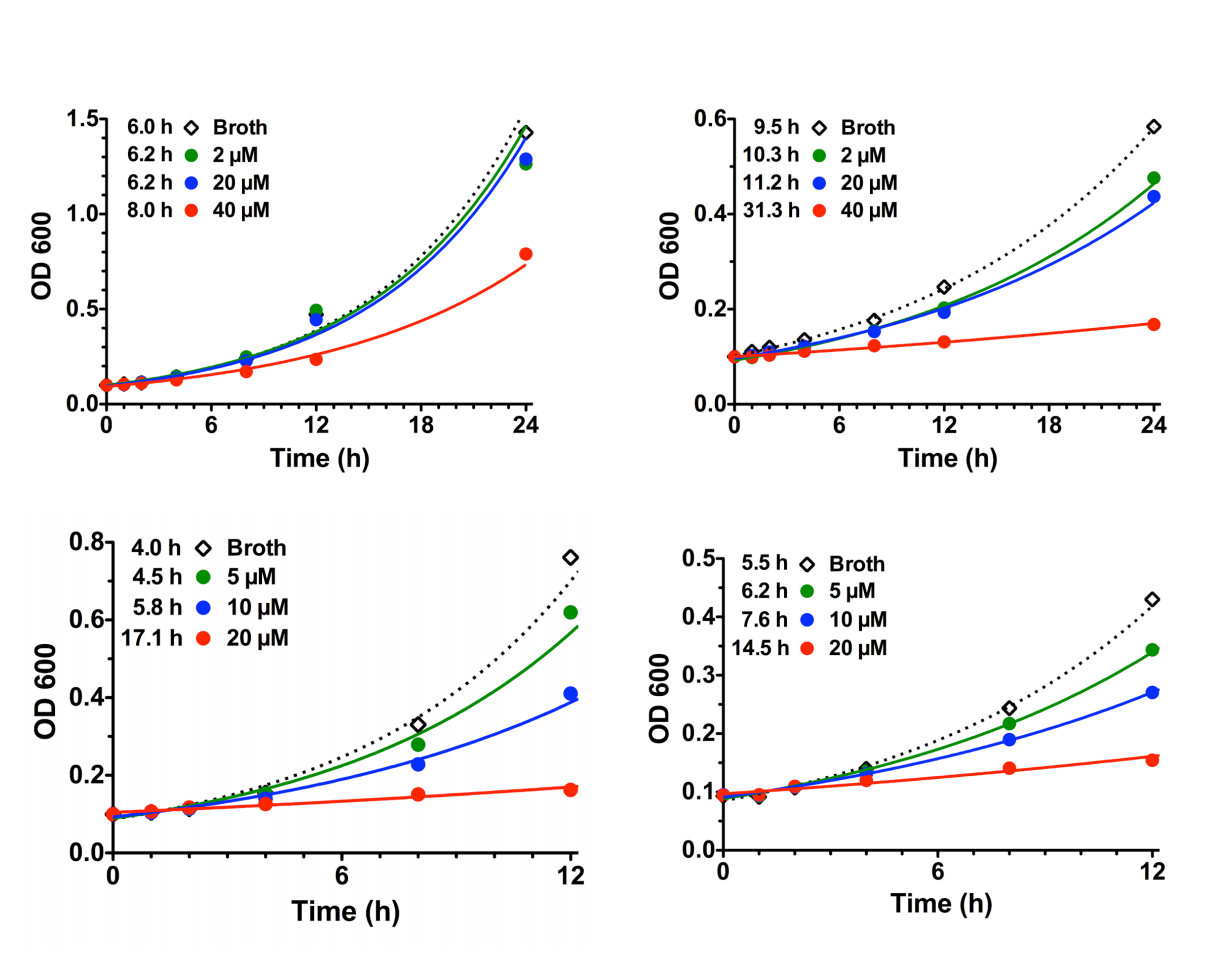


**Supplementary Figure. 1. Generation time of different strains of *H. pylori* treated with curcumin or Meriva.** (A,B) *H. pylori* representative strains 7.13 and 26695 were grown in brucella broth with indicated concentration of curcumin and growth was monitored for indicated time. (C, D*). H. pylori* representative strains PZ5056G and PMSS1 were grown in brucella broth with indicated concentration of Meriva and growth was recorded at indicated time points.

**
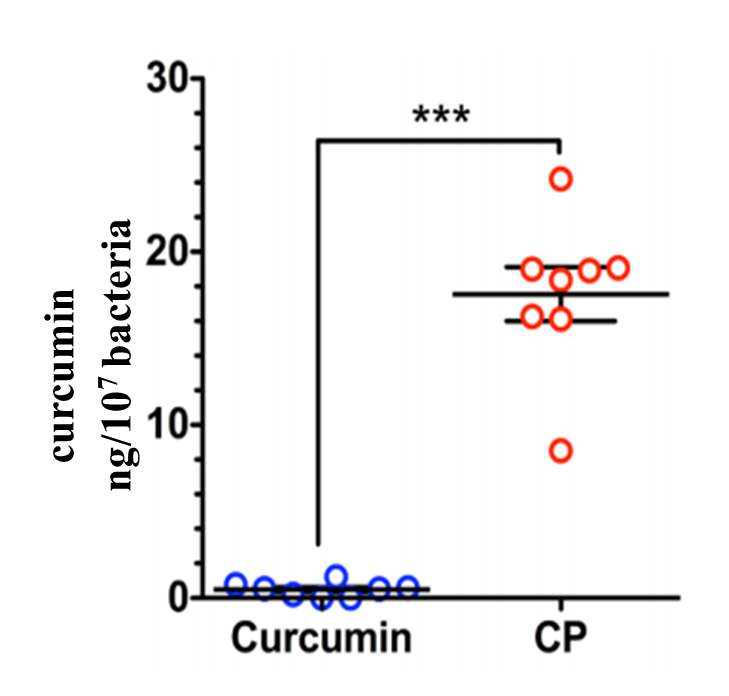
**

**Supplementary figure.** 2. **Curcumin uptake by *H. pylori*.** The intracellular accumulation of curcumin in *H. pylori* treated with synthetic curcumin or Meriva (CP) (****P* < 0.0005).

**
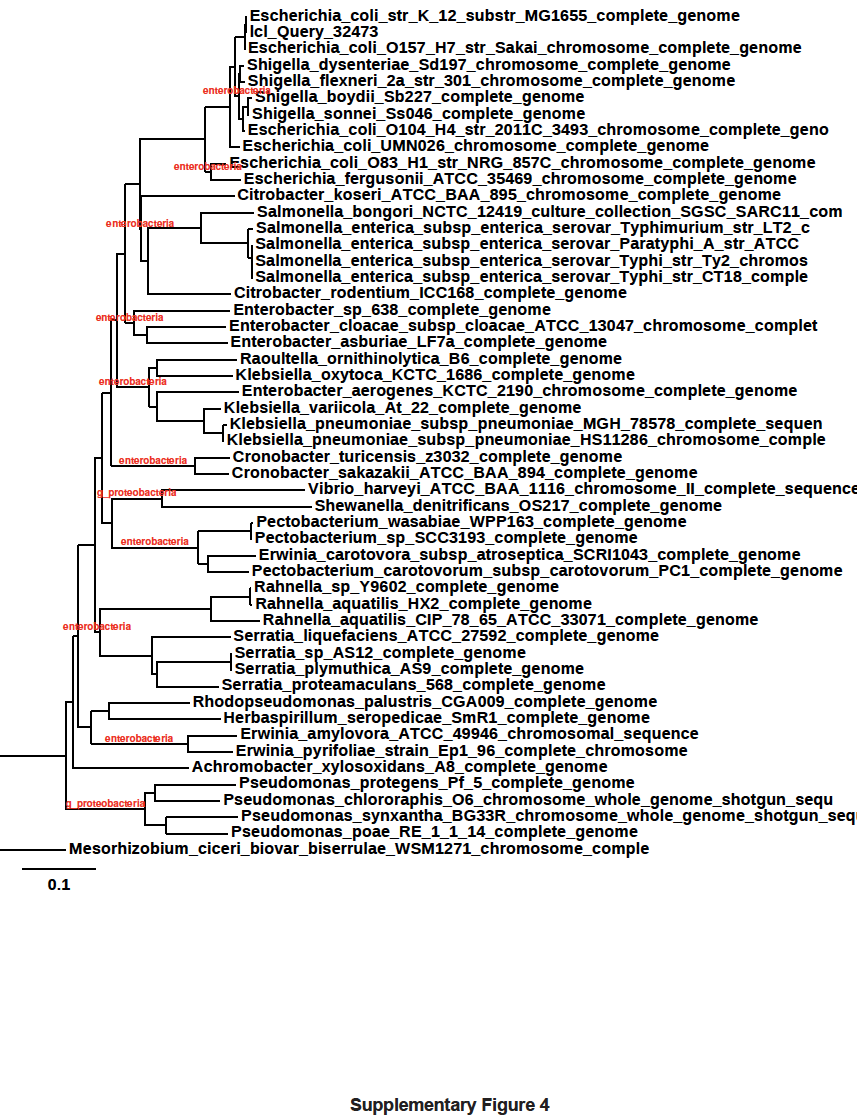
**

**Supplementary Figure. 3.** Phylogenetic tree constructed from the nucleotide sequences of *curA* genes of enteric bacteria. The scale bar corresponds to 0.1 change per nucleotide.


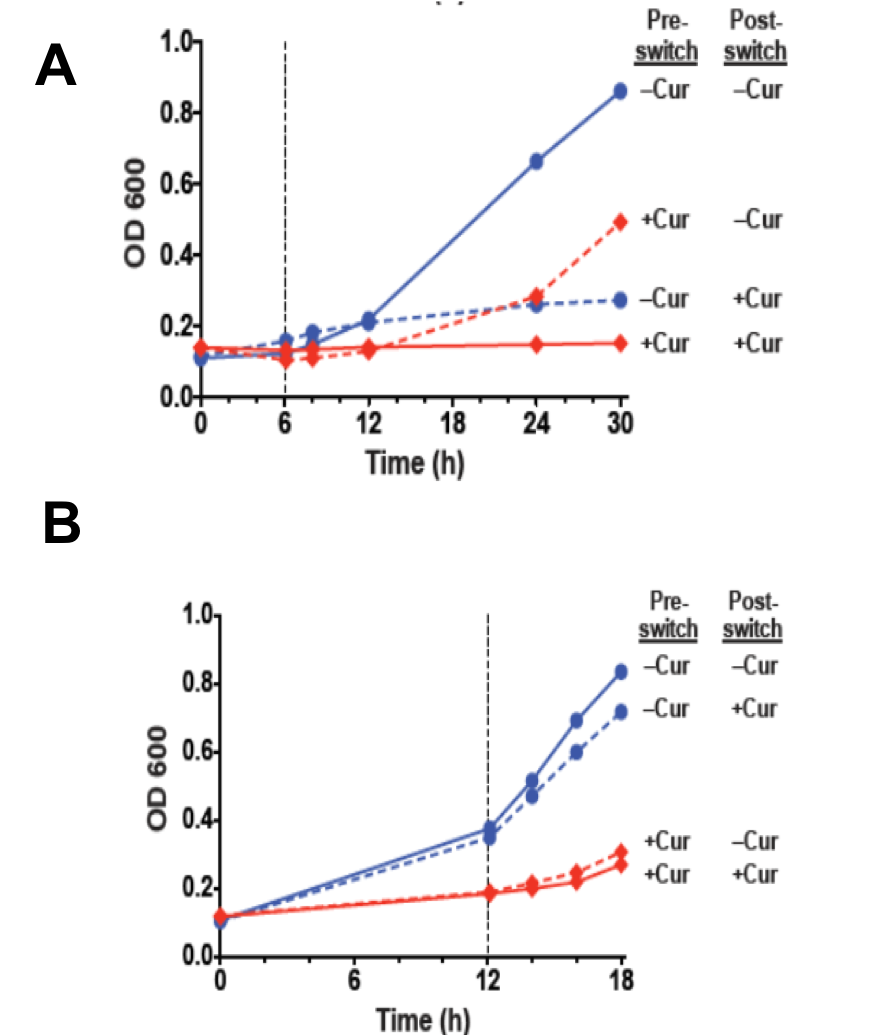


**Supplementary Figure 4. Switching in growth experiment of *H. pylori* in Meriva.** (A) Growth curves of *H. pylori* were generated by growing bacteria in brucella broth with 10% fetal bovine serum (FBS). *H. pylori* was grown for 6 h in broth medium containing 20 μM Meriva and then transferred to broth without Meriva. (B). Growth curves of *H. pylori* were generated by growing bacteria in brucella broth with 10% fetal bovine serum (FBS). *H. pylori* was grown for 12 h in the broth containing Meriva, bacteria did not recover when transferred to broth medium.

**Mouse 2**

**Mouse 1**

**
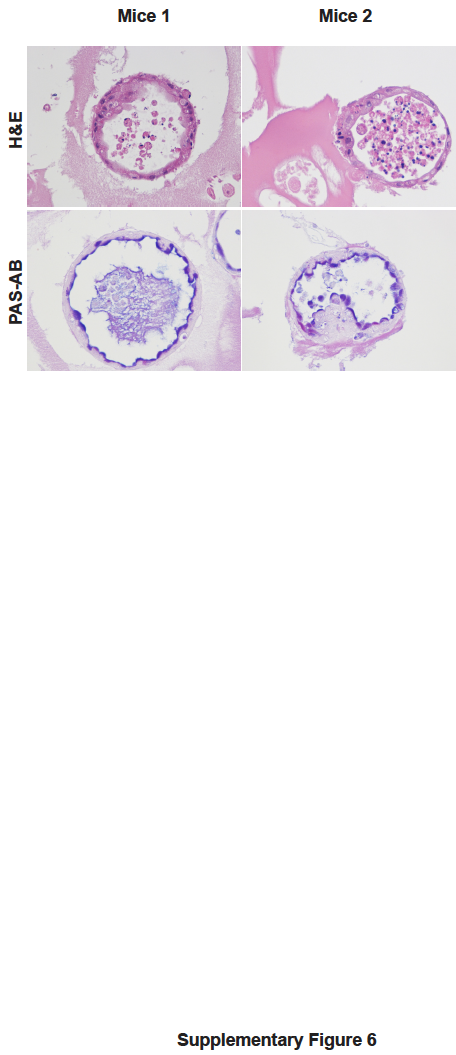
**

**Supplementary Figure 5. Histology of mouse gastric organoids.** Mouse stomach glands were isolated by ligating at the esophago-gastric and gastro-duodenal junctions. Released glands were plated in Matrigel. Gastroids were cultured in advanced DMEM/F12 medium without penicillin/streptomycin. Some of the wells were fixed in 4% formaldehyde and embedded in paraffin blocks. 5 μM thin sections were cut and gastroids were stained with H&E and also for PAS-AB.


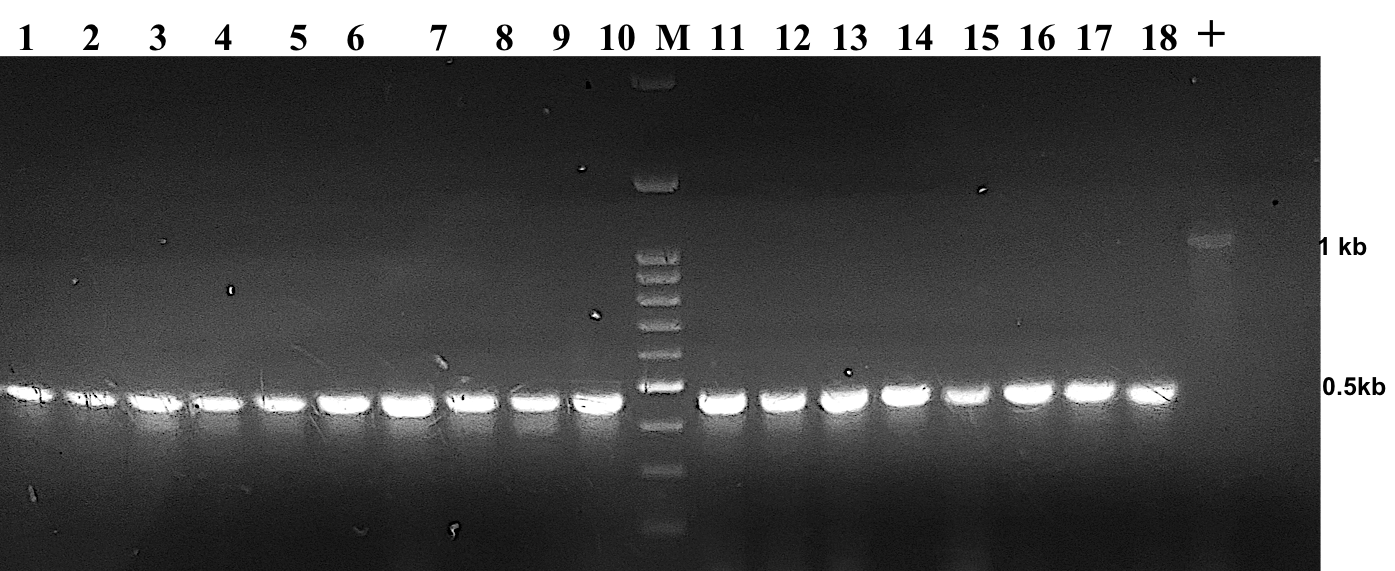


**Supplementary figure 6.** Agarose gel electrophoresis of PCR amplified products using *cur A* and *ureaA*  PCR primer sets. Lanes 1–18 loaded with both *curA*  and urease gene (internal control) amplified products in equal concentration. No band observed near size of curA in any clinical isolates. Size of curA 1 kb and size of urease 428. Lane + positive control (*E coli*), M, 100bp DNA size marker.

Chaturvedi, R., Asim, M., Hoge, S., Lewis, N.D., Singh, K., Barry, D.P., de Sablet, T., Piazuelo, M.B., Sarvaria, A.R., Cheng, Y., Closs, E.I., Casero, R.A., Jr., Gobert, A.P., Wilson, K.T., 2010. Polyamines Impair Immunity to Helicobacter pylori by Inhibiting L-Arginine Uptake Required for Nitric Oxide Production. Gastroenterology 139, 1686-1698, 1698.e1681-1686.

Rosen, M.J., Chaturvedi, R., Washington, M.K., Kuhnhein, L.A., Moore, P.D., Coggeshall, S.S., McDonough, E.M., Weitkamp, J.H., Singh, A.B., Coburn, L.A., Williams, C.S., Yan, F., Van Kaer, L., Peebles, R.S., Jr., Wilson, K.T., 2013. STAT6 deficiency ameliorates severity of oxazolone colitis by decreasing expression of claudin-2 and Th2-inducing cytokines. Journal of immunology (Baltimore, Md. : 1950) 190, 1849-1858.
